# Supplementary material for: Optic Nerve Crush Does not Induce Retinal Ganglion Cell Loss in the Contralateral Eye
Source: Invest Ophthalmol Vis Sci. 2025 Mar 24;66(3):49. doi: 10.1167/iovs.66.3.49 (PMC11951053; doi:10.1167/iovs.66.3.49)
Supplement: Supplement 1 [file iovs-66-3-49_s001.pdf]

Figure S1.

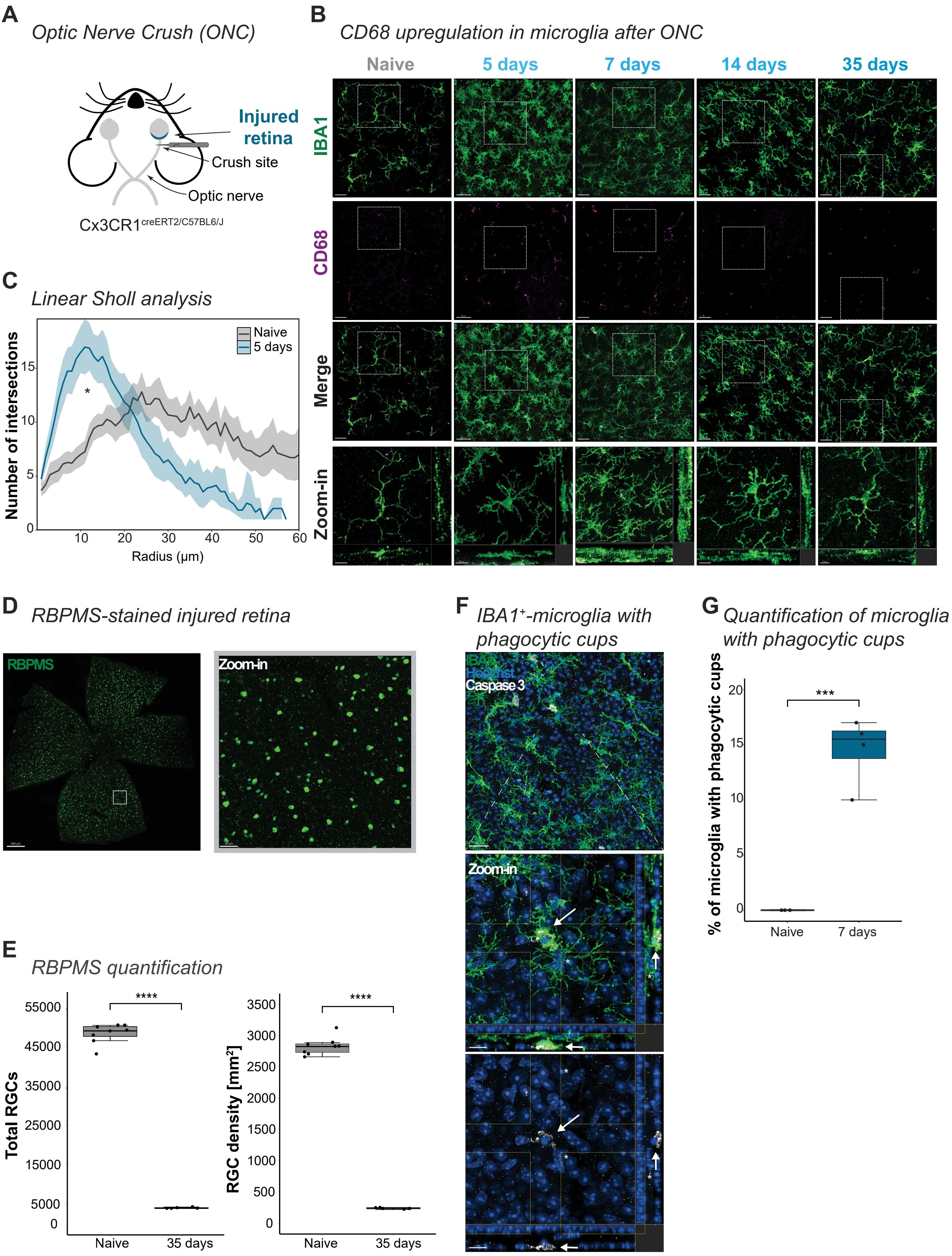
A
Optic Nerve Crush (ONC)
Injured retina
Crush site
Optic nerve
Cx3CR1creERT2/C57BL6/J
B
CD68 upregulation in microglia after ONC
Naive
5 days
7 days
14 days
35 days
IBA1
CD68
Merge
Zoom-in
C
Linear Sholl analysis
Naive
5 days
Number of intersections
Radius (µm)
D
RBPMS-stained injured retina
RBPMS
Zoom-in
E
RBPMS quantification
Total RGCs
RGC density [mm²]
Naive
35 days
F
IBA1+-microglia with phagocytic cups
Hoechst
Caspase 3
Zoom-in
G
Quantification of microglia with phagocytic cups
% of microglia with phagocytic cups
Naive
7 days

Figure S1. Microglial response and RGC death in the injured eye after optic nerve crush
(A) Schematic of optic nerve crush (ONC).
(B) Representative maximum intensity projection images of the retinal inner plexiform layer (IPL) immunostained for IBA1 (microglia, green) and CD68 (magenta) in naïve or in the injured eye 5, 7, 14, or 35 days after ONC. Scalebar: 20 µm, zoom-in with orthogonal projection: 10 µm.
(C) Mean number of Sholl interactions per radial distance from the soma (µm) with 95% confidence interval band naive (n= 16 microglia from 6 retinas) and 5 days after ONC (n= 22 microglia from 8 retinas) in the injured eye. Linear mixed effect model: p= 0.0327.
(D) Representative image of RBPMS-stained retinal wholemount 35 days after ONC. Scale bar: 500 µm, zoom-in: 40 µm.
(E) Box plots of total count and density of RBPMS^{+}-cells. Student's t-test: $p_{total\ RGC\ count}$=3.28×10-13, $p_{RGC\ density}$=112.85×10-13.
(F) Representative image of IBA1^{+}-microglia in the retinal ganglion cell layer of the injured retina, 7 days after ONC. Arrow: phagocytic cup. Asterisk: cell body of microglia. Scalebar: 20 µm, zoom-in with orthogonal projection: 10 µm.
(G) Quantification of the percentage of microglia with phagocytic cups in the field of view. Student's t-test: p= 0.000426.
For detailed statistical analysis, see **Supplementary Table 1**. ****p<0.0000. ***p<0.001. *p < 0.05.
