# Supplementary material for: Optic Nerve Crush Does not Induce Retinal Ganglion Cell Loss in the Contralateral Eye
Source: Invest Ophthalmol Vis Sci. 2025 Mar 24;66(3):49. doi: 10.1167/iovs.66.3.49 (PMC11951053; doi:10.1167/iovs.66.3.49)
Supplement: Supplement 2 [file iovs-66-3-49_s002.pdf]

Figure S2.

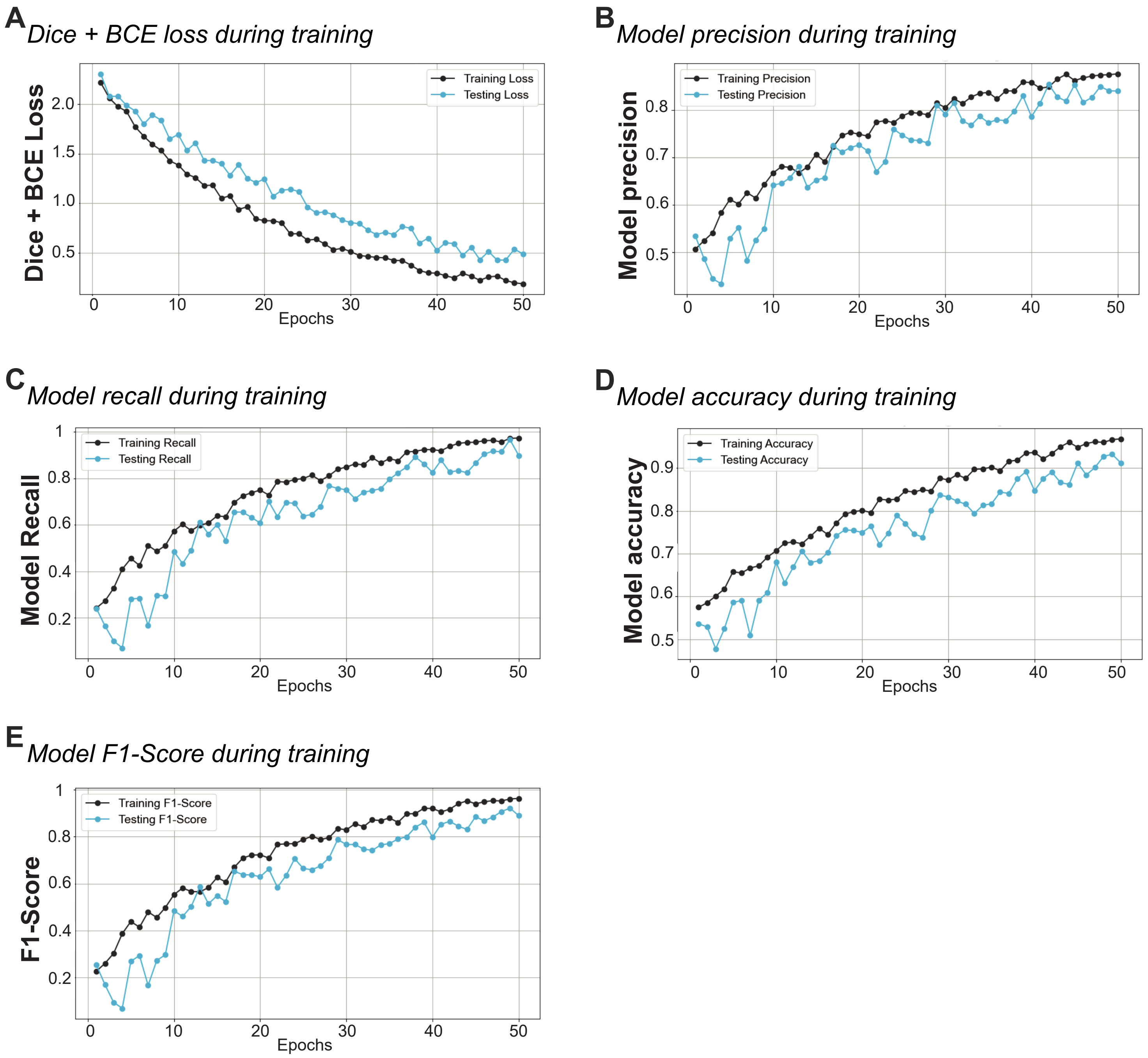

Figure S2. Training of RGC-Quant algorithm

(A) Loss over epochs for training and testing datasets. The loss decreases as the number of epochs, one complete pass of the training dataset through the algorithm, increases, indicating model convergence.
BCE: binary cross entropy.
(B) Precision over epochs for training and testing datasets. Precision improves as the model learns to minimize false positives.
(C) Recall over epochs for training and testing datasets. Recall improves as the model learns to minimize false negatives.
(D) Accuracy over epochs for training and testing datasets. Accuracy improves as the model learns to classify more samples correctly.
(E) F1-Score over epochs for training and testing datasets. The F1-score, which balances precision and recall, improves as the model continues training.
