# Supplementary material for: Optic Nerve Crush Does not Induce Retinal Ganglion Cell Loss in the Contralateral Eye
Source: Invest Ophthalmol Vis Sci. 2025 Mar 24;66(3):49. doi: 10.1167/iovs.66.3.49 (PMC11951053; doi:10.1167/iovs.66.3.49)
Supplement: Supplement 4 [file iovs-66-3-49_s004.pdf]

Supplementary Table 2

Method	Animal model	Timepoint measured	Distance from optic disk	RGC labeling	Contralateral RGC loss	References
ONT	Brown-Norway rats	3 weeks	1 mm	Cholera toxin beta	yes	Choe, *et al.,* (2014). *PLoS ONE*[22]
IOP	Brown Norway rats	14 days	Not reported	RBPMS	no	Tribble, *et al.,* (2021). *Translational Vision Science and Technology*[35]
ONC	BALB/cJ mice	28 days	1 mm	Nissl	yes	Liu, *et al.,* (2014). *Investigative phthalmology and Visual Science*[23]
ONT	Sprague-Dawley rats	4 months	0.5 mm	Brn3a	no	Rovere, *et al.,* (2015). *Investigative Ophthalmology and Visual Science*[21]
ONC	Sprague Dawley rats	6 months	3 mm	Brn3a	No	Nadal-Nicolás, *et al.,* (2015). *Investigative Ophthalmology and Visual Science*[19]
ONT	Sprague Dawley rats	15 months	0.5 mm	Brn3a	no	Nadal-Nicolás, *et al.,* (2015). *Investigative Ophthalmology and Visual Science*[19]
ONC	C57BL6/J mice	3 months	1 mm	nuclear dye To-Pro1	no	Qu, & Jakobs, (2013). *PLoS ONE*[20]
ONC	C57BL6/J mice	9, 45 days	0.5 mm, 2 mm	Brn3a	yes	Lucas-Ruiz, *et al.,* (2019). *International Journal of Molecular Sciences*[24]
ONT	Swiss, C57/BL6 mice	7 days, 14 days	0.5 mm	Brn3a	Not significant	Galindo-Romero, et al., (2013). *Investigative Ophthalmology and Visual Science*[34]
IOP	Long Evans rats	6 weeks	Not reported	Brn3a	Not significant	Gramlich, *et al.,* (2016). *Journal of Neuroinflammation*[43]
